# Involvement of Visceral Adipose Tissue in Immunological Modulation of Inflammatory Cascade in Preeclampsia

**DOI:** 10.1155/2015/325932

**Published:** 2015-05-31

**Authors:** Katsuhiko Naruse, Juria Akasaka, Aiko Shigemitsu, Taihei Tsunemi, Natsuki Koike, Chiharu Yoshimoto, Hiroshi Kobayashi

**Affiliations:** Department of Obstetrics and Gynecology, Nara Medical University, Kashihara, Nara 6348521, Japan

## Abstract

*Objectives*. The pathophysiology of preeclampsia is characterized by abnormal placentation, an exaggerated inflammatory response, and generalized dysfunction of the maternal endothelium. We investigated the effects of preeclampsia serum on the expression of inflammation-related genes by adipose tissue. *Materials and Methods*. Visceral adipose tissue was obtained from the omentum of patients with early ovarian cancer without metastasis. Adipose tissue was incubated with sera obtained from either five women affected with severe preeclampsia or five women from control pregnant women at 37°C in a humidified incubator at 5% CO_2_ for 24 hours. 370 genes in total mRNA were analyzed with quantitative RT-PCR (Inflammatory Response & Autoimmunity gene set). *Results*. Gene expression analysis revealed changes in the expression levels of 30 genes in adipose tissue treated with preeclampsia sera. Some genes are related to immune response, oxidative stress, insulin resistance, and adipogenesis, which plays a central role in excessive systemic inflammatory response of preeclampsia. In contrast, other genes have shown beneficial effects in the regulation of Th2 predominance, antioxidative stress, and insulin sensitivity. *Conclusion*. In conclusion, visceral adipose tissue offers protection against inflammation, oxidative insults, and other forms of cellular stress that are central to the pathogenesis of preeclampsia.

## 1. Introduction

Preeclampsia is the leading cause of pregnancy-associated maternal and perinatal mortality and morbidity worldwide. This disorder affects approximately 5% of all pregnancies. Several mechanisms have been proposed in preeclampsia, including (1) genetics and epigenetic imprinting; (2) increased uteroplacental ischemia/hypoxia; (3) angiogenic imbalances characterized by an excess of antiangiogenic factors; (4) increased trophoblast apoptosis/necrosis; (5) an exaggerated maternal inflammatory response to injured trophoblast cells; and (6) immune maladaptation [1]. Shallow trophoblast invasion and inadequate artery remodeling early in pregnancy may underlie subsequent placental hypoperfusion, hypoxia, or ischemia, which are critical components in the pathogenesis of preeclampsia [2]. Maternal responses are associated with release of placenta-derived circulating antiangiogenic molecules such as soluble fms-like tyrosine kinase 1 (sFlt-1 or the soluble VEGF receptor-1), soluble endoglin (sEng), the angiotensin II type-1 receptor autoantibody (AT1-AA), and proinflammatory cytokines such as tumor necrosis factor- (TNF-) alpha and interleukin- (IL-) 6 [3]. The loss of endothelial control of vascular development by these factors in turn acts in concert to cause hypertension and decrease renal function during pregnancy [3]. Placenta-derived circulating factors could also stimulate proinflammatory cells to produce cytokines and chemokines, including IL-1beta, IL-2, IL-10, IL-12, IL-13, IL-18, granulocyte-colony stimulating factor (G-CSF), interferon- (IFN-) gamma, monocyte chemoattractant protein-1 (MCP-1), and TNF-alpha, demonstrating that preeclampsia is associated with an overall proinflammatory systemic environment [4–6]. Although a normal pregnancy enhances a state of the T helper 2 (Th2) type anti-inflammatory responses, preeclampsia exhibits a shift towards Th1 [4, 7] and Th17 [8] type immunity.

The discovery of biologically functional numerous proinflammatory, anti-inflammatory, and immunomodulating proteins, cytokines, and chemokines in adipocytes emphasizes the role of the adipose tissue as a highly active immune response, endocrine and metabolically important organ that modulates energy expenditure, insulin resistance, and glucose homeostasis [9, 10]. Adipose tissue is capable of contributing to this inflammation by its production of inflammatory mediators, which appears to be a key step in the development of the preeclampsia-associated inflammatory state.

Here, we aim to investigate whether preeclampsia sera could modulate the inflammatory and adipogenic activities in visceral adipose tissue, using new method of the tissue culture. Our data suggest a revised paradigm for restoring host defense and preventing inflammatory sequelae in adipose tissue in women affected with preeclampsia.

## 2. Materials and Methods

### 2.1. Sample Collection

The study was approved by the Local Ethics Committee at Nara Medical University, and all participants provided written informed consent. Visceral fat (omentum) was taken from two women at reproductive age (36 years old and 40 years old) who underwent prophylactic omentectomy on ovarian cancer operation. No metastasis of cancer or inflammation was confirmed with pathological search. The tissue was immediately suspended in cold sterile saline, transported to the laboratory, washed several times in sterile phosphate buffered saline to remove excess blood, and dissected to twenty pieces of approximately 1.5 g.

Next, we included severe preeclampsia (PE) patients of with a prepregnancy body mass index (BMI) before pregnancy was under less than 25 kg/m^2^ with gestational age-matched normal pregnant women at 28 weeks gestation or later. All subjects of them were Eastern Asian origin, and none of the subjects were taking any medication or showed evidence of any metabolic disease or other complications beside PE. Severe PE was defined as new onset and diagnosed based on two consecutive measurements of diastolic and systolic blood pressure measurements, diastolic blood pressure greater than or equal to 110 mmHg, or systolic blood pressure ≥160 mmHg, respectively, with urine protein over 2 g/day, occurring diagnosed after 20 weeks of gestation [11]. All subjects had provided serum samples available for analysis and did not have gestational diabetes mellitus, thyroid malfunction, or other complications. Briefly, 5 women with severe preeclampsia with BMI ranging from 22.6 to 25.2 kg/m^2^ at test and 5 age- and BMI-matched control pregnant women were recruited.

Characteristics of the subjects serum taken were shown in Table 1.

### 2.2. Whole Adipose Tissue Culture

In this study, we established new method for bottom culture of whole adipose tissue, not only adipocyte but other cells and connective tissue as well. Dissected visceral fat was captured immediately on the bottom of the 24 well plastic plate (Becton, Dickinson & Co., Franklin Lakes, NJ) with 99.5% medium-containing hydrogel (PuraMatrix, Becton, Dickinson & Co.) after provider's manual. After enough fiber construction, culture medium (Eagle's minimal essential medium, Sigma-Aldrich Co., St. Louis, MO) without any serum was added and changed for three times to adjust medium's pH as homeostatic range. The tissue was starved for 12 hours until next process.

Next day, serum from PE or healthy pregnant subjects (*n* = 5 each) was added in 1 : 10 order in the wells in duplicate. The human serum concentrations in the medium were decided from former report for bovine serum concentrations in the culture medium treating mice adipose tissue and separated cells [12]. After 24 hours of culture under incubator of 37°C/21% O_2_/5% CO_2_ condition, medium and the tissue were collected. All adipose tissues are stock-frozen immediately with liquid nitrogen until mRNA extraction.

### 2.3. mRNA Extraction and Profiler Array

Total mRNA from visceral fat was extracted at Genetic Lab Co., Ltd. (Sapporo, Japan). The purity of mRNA was confirmed with OD260/280 (range: 2.07–2.11) and RNA Integrity Number (range: 7.5–8.3). And then, 370 genes in total mRNA were analyzed with quantitative RT-PCR (Inflammatory Response & Autoimmunity gene set, RT2 Profiler PCR Array, Qiagen Inc., Germany).

### 2.4. Statistical Analysis

Results of the quantitative RT-PCR were shown as fold change on PE serum-added adipose tissue against normal serum added tissue after being normalized with internal control gene (beta-2-microglobulin (B2M), hypoxanthine phosphoribosyltransferase 1 (HPRT1), ribosomal protein L13a (RPL13A), glyceraldehyde-3-phosphate dehydrogenase (GAPDH), actin, beta (ACTB)) expression. The criteria for selecting differentially expressed genes were preset as at least 2-fold difference in either direction or genes with statistical significance (*P* < 0.05, unpaired *t*-test). Statistical calculations were performed using SPSS 15.0J (SPSS Japan Inc., Japan) on each gene with Student's *t*-test comparing PE and the normal (*n* = 5 each), with *P* < 0.05 indicating a statistically significant difference.

## 3. Results

### 3.1. Identification and Functional Classification of Differentially Expressed Genes

**Figure 1:**
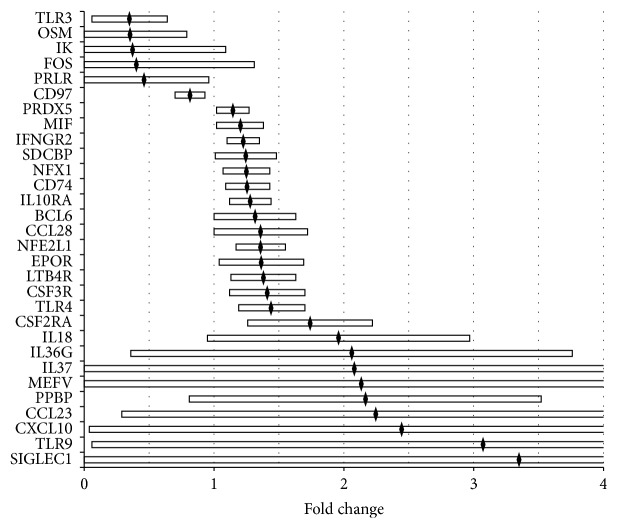
Genes identified with altered expression in adipose tissues treated with preeclampsia sera. Thirty genes showed alteration at least 2-fold or statistical significance (*P* < 0.05, unpaired *t*-test). Black diamond shows mean and open bars show 95% CI of respective genes.

Thirty genes were identified with altered expression of at least 2-fold or statistical significance (*P* < 0.05, unpaired *t* test) in adipose tissues treated with preeclampsia sera (Figure 1, Table 2). Among these genes, the greatest up- or downregulation was observed in genes involved in immune response, oxidative stress, and insulin and lipid metabolism, any of which may contribute to the molecular mechanisms underlying insulin resistance and adipogenesis in preeclampsia (Table 3). Our results show that exposure of preeclampsia sera increased or decreased the expression of several genes and affected the functional pathways, including (1) energy balance, obesity, lipid metabolism, and adipogenesis, (2) insulin resistance and glucose tolerance, (3) host defense, redox balance, detoxification, and oxidative stress, and (4) inflammation, immune response, and Th1/Th2 type cytokine balance. See Supplementary data  in Supplementary Material available online at http://dx.doi.org/10.1155/2015/325932.

### 3.2. Genes Involved in Immune Response

Microarray analysis identified 11 upregulated and 2 downregulated immune response-related genes in preeclampsia sera-stimulated adipose tissue. Interestingly, Th1/Th2 type cytokine and immune responsive genes were significantly regulated. RT-qPCR confirmed changes in expression of Th1 type cytokine-related genes (IL18, CXCL10, and IK) and Th2 type cytokine-related genes (BCL6, CCL28, LTB4R, and IL27), with Th2/Th1 predominance. Differential expression of Th17-related cytokine (IL36G) and other immune responsive genes (MEFV, PPBP, CCL23, SIGLEC1, and CD97) was also confirmed by RT-qPCR and independently validated.

### 3.3. Genes Involved in Oxidative Stress

Oxidative stress signaling genes were also differentially expressed. Among oxidative stress-related genes, 7 genes (PRDX5, MIF, CD74, NFE2L1, CSF3R, TLR4, and TLR9) were induced while no genes were suppressed. RT-qPCR confirmed aberrant expression of genes involved in inflammation and stress response (TLR4 and TLR9) and also genes involved in host defense (PRDX5, MIF, CD74, NFE2L1, and CSF3R), suggesting that preeclampsia sera suppress the TLR4/9-dependent excess oxidative stress in adipose tissue.

### 3.4. Genes Involved in Glucose and Lipid Metabolism

Our results indicated that 6 upregulated genes (IFNGR2, NFX1, IL10RA, SDCBP, EPOR, and CSF2RA) and 4 downregulated genes (TLR3, FOS, PRL, and OSM) were involved in glucose and lipid biosynthesis (Table 3). The amount of insulin resistance genes (IFNGR2 and NFX1) may correlate with the preeclampsia syndrome, which is regarded as a key feature of preeclampsia genesis. In contrast, four genes such as IL10RA, TLR3, FOS, and PRL demonstrate strong inverse correlations with insulin resistance.

We also identified four genes associated with adipogenesis, indicating that the SDCBP and OSM genes stimulate adipogenesis, while EPOR and CSF2RA significantly reduce it. Adipose tissue produced several genes in the homeostasis of glucose and lipid metabolism as well as adipogenesis.

On the one hand, preeclampsia sera expectedly enhance inflammatory activities, including immune response, oxidative stress, insulin resistance, and adipogenesis, in adipose tissue. On the other hand, they can also suppress inflammation through upregulation of Th2 cytokine predominance, antioxidative stress, and insulin sensitivity. These data collectively support that visceral fat in women affected with preeclampsia might promote and strongly suppress the inflammatory and adipogenic activities.

## 4. Discussion

Preeclampsia is strongly associated with abnormal placentation characterized by shallow trophoblast invasion and incomplete spiral artery remodeling, which causes elevated amounts of proinflammatory cytokines, chemokines, adhesion molecules, and growth factors [4]. Chronic inflammation and endothelial injury might play a central role in the pathogenesis of preeclampsia, but the underlying pathophysiology is still unclear. We demonstrate significant differences in gene expression of adipose tissue treated with sera from nonobese preeclampsia patients and age- and BMI-matched controls. Adipose tissue might be contributing to modulation of the potential functional genes for inflammation and immune response (Th1/Th2 predominance), oxidative stress, insulin resistance, and adipogenesis (Table 3). The altered gene products in adipose tissue lead to suppression of preeclampsia-associated inflammation which in turn is responsible for excessive production of Th2 type cytokines and host defense molecules, as well as modulation of adipogenesis and insulin resistance.

These data allow us to hypothesize that when chronic inflammation is consistently present, anti-inflammation remains active in adipose tissue. In an early event of placental dysfunction, newly synthesized inflammatory cytokines and chemokines drive association of inflammation and oxidative stress, leading to insulin resistance and adipogenesis. The second wave of preeclampsia supports sustained expression of a subset of inhibition of oxidative stress, insulin resistance, and adipogenesis in adipose tissue, several of which play important roles in preeclampsia. This study reveals new aspects of preeclampsia and adipocyte biology.

Firstly, RT-qPCR data demonstrated significant differences in immune response gene expression. These genes are immunomodulators induced under stressful or pathological conditions such as preeclampsia. Pregnancy is associated with Th2 type cytokine predominance or downregulation of the Th1 response, which is more pronounced at the maternal fetal interface [13]. Th1 cells produce an array of proinflammatory cytokines including IFN-gamma, IL-2, and TNF-alpha. Th2 cells produce IL-4, IL-5, and IL-10. The majority of publications report on aberrant Th1/Th2 balance and upregulation of the Th17 immune response in preeclampsia [14]. We for the first time confirmed that preeclampsia sera could induce changes of Th1/Th2 cytokine balance with a predominance of Th2 immunity in adipose tissue, suggesting the role of immunological mechanisms engaged in preeclampsia. After the establishment of preeclampsia, predominance might be shifted from Th1 cells to Th2 cells in adipose tissue.

Secondly, altered expression was observed in several defense and stress response genes associated with oxidative stress, which is involved in regulating host defense. It appears that preeclampsia is a disease of exaggerated innate immunity that may be mediated by Toll-like receptors (TLRs). Previous studies have also identified immune-system alterations associated with the origin of preeclampsia as well as genetic associations between TLRs and preeclampsia: TLR2 and TLR4 SNPs appear to alter susceptibility to developing preeclampsia [15]. This study showed that preeclampsia sera stimulate expression of TLR4 and TLR9 in adipose tissue. TLR4 generates local and systemic inflammatory and oxidative stress responses in preeclampsia [16]. Oxidative stress can in turn induce and maintain inflammatory responses mainly through a TLR4-dependent nuclear factor- (NF-) kappaB pathway [17]. Exaggerated placental cell injury and death result in the release of mitochondrial DNA, which activates TLR9 to produce systemic maternal inflammation from adipocytes, and subsequent vascular dysfunction that may in turn lead to preeclampsia [18]. TLR9 and IFN-gamma were located in differentiated and mature adipocytes [19]. The TLR4 and TLR9 activation in adipose tissue may worsen the situation of patients with preeclampsia. In contrast, we identified increased expression levels of 5 genes such as PRDX5, MIF, CD74, NFE2L1, and CSF3R, which play an essential role in the host immune response or the host defense against several pathogens or oxidative stress. It is possible that increased expression of these genes in adipose tissue could strengthen host defense by protecting host cells from oxidative insults.

Thirdly, genes involved in insulin resistance are differentially expressed in adipose tissue stimulated with preeclampsia sera. It has been established that women with preeclampsia have an increased risk of developing diabetes [20]. Although insulin resistance is a key pathophysiology of preeclampsia, the mechanisms remain unclear. Our data demonstrated significant increases in the expression levels of several lipid metabolism-related genes, including IFNGR2 and NFX1, which modulate lipid metabolism to promote insulin resistance. In contrast, we identified decreased expression of selected genes involved in insulin resistance in adipose tissue, including TLR3, FOS, and PRL, which could induce insulin sensitivity. IL10RA is also negatively involved in insulin resistance. Thus, preeclampsia sera might contribute to insulin sensitivity by positive and negative regulation of the expression of diverse genes.

Finally, adipose tissue is a highly active endocrine and metabolically important organ, with the ability to modulate glucose homeostasis, energy expenditure, lipid metabolism, and peripheral inflammation. Our results identified increased expression of selected genes involved in lipid metabolism, including SDCBP, EPOR, and CSF2RA. Increased expression of SDCBP in adipocytes likely contributes to adipogenesis, whereas EPOR and CSF2RA are negatively involved in adipogenesis [21–23]. EPOR regulates energy homeostasis and mitigates adipogenesis via the metabolism coregulators peroxisome proliferator-activated receptor alpha (PPARalpha) and sirtuin 1 (Sirt1) [21–23]. Furthermore, CSF2RA is a receptor for CRF2, also known as GM-CSF, which is related to a central action to reduce food intake and body weight, since knockout mice are more obese and hyperphagic than wild-type mice [24]. OSM inhibits the terminal differentiation of adipocytes through the Ras/extracellular signal-regulated kinase (ERK) and signal transducer and activator of transcription (STAT) 5 signaling pathways [25, 26]. Preeclampsia sera inhibited the OSM gene expression in adipose tissue. Therefore, preeclampsia sera could relieve insulin resistance and adipogenesis in adipose tissue.

Over the last decade preeclampsia biology revealed that the early molecular changes affect inflammation, immune response, angiogenesis, oxidative stress, matrix remodeling, and lipid biosynthesis [27]. The TLR signaling pathway induces inflammation, which in turn modulates insulin resistance and adipogenesis [28, 29]. Inflammation, oxidative stress, insulin resistance, and adipogenesis, secondary to the influx of proinflammatory cytokines and chemokines during placental dysfunction, are involved in the progression of preeclampsia. Preeclampsia serum priming in adipose tissue leads to enhanced Th1 inflammation, oxidative stress, and insulin resistance, and simultaneously antiadipogenic induction may result in enhanced expression of Th2 predominance, antioxidative stress, and insulin sensitivity.

This study limits the ability to ascribe causality to the association between adipocytes and their gene expression. Adipose tissue used in this study contains adipocytes, macrophages, T lymphocytes, other immune cells, vasculature, and stromal cells. Further study will be conducted to confirm the anti-inflammatory effects of preeclampsia serum and to elucidate its mechanism of action in adipocytes in culture.

In conclusion, this study supports the hypothesis that there are at least two distinct phases of preeclampsia development: the initial wave of inflammatory activation in modulating immune response, oxidative stress, insulin resistance, and adipogenesis would be followed by the second big wave of anti-inflammation in adipose tissue. Finally, adipose tissue may have an ability to suppress inflammation, immune response, oxidative stress, and metabolic signals to protect host from excessive inflammation.

## 5. Conclusions

The primary event in the molecular sequence leading to chronic inflammation is placental dysfunction in preeclampsia. Increased inflammation likely contributes to adipokine dysregulation, adipogenesis, and insulin resistance in adipose tissue. This initial wave of the systemic inflammation would be followed by the second big wave of subsequent production of anti-inflammatory mediators by adipose tissue, which then suppresses oxidative stress, insulin resistance, and metabolic dysfunction. Adipose tissue may protect host from excessive inflammation in preeclampsia.

## Supplementary Material

Supplementary data shows the detailed explanation of genes differentially expressed in adipose tissue treated with nonobese preeclampsia sera versus matched control sera.

## Figures and Tables

**Table 1:** Characteristics of the study population of the sera.

	Normal pregnancy	Preeclampsia
*n*	5	5
Maternal age at sampling (years)	31.8 ± 2.1	31.5 ± 2.9
Gestational age at sampling (weeks^+days^, range)	28^+0^ (27^+1^–28^+4^)	29^+1^ (26^+5^–31^+3^)
BMI at sampling (kg/m^2^)	24.1 ± 1.6	23.5 ± 0.5
BMI before pregnancy (kg/m^2^)	22.7 ± 2.3	20.6 ± 0.4
Blood pressure (mm/Hg)		
Systolic	121.2 ± 1.9	182.8 ± 5.7^*∗*^
Diastolic	67.8 ± 1.9	117.2 ± 3.4^*∗*^
MAP	85.6 ± 1.7	139.1 ± 3.9^*∗*^

BMI: body mass index; MAP: mean arterial pressure. All patients in preeclampsia group showed urine protein over 2 g/day. Data has shown as mean ± S.E.M. unless indicated.

^*∗*^ *P* < 0.05 versus normal pregnancy.

**Table 2:** Genes identified with altered expression in adipose tissues treated with preeclampsia sera.

Symbol	Fold change	95% CI	*P*-value
TLR3	**0.348 **	(0.06, 0.64)	**0.016 **
OSM	**0.353 **	(0.00001, 0.79)	**0.019 **
IK	**0.372 **	(0.00001, 1.09)	*0.347 *
FOS	**0.400 **	(0.00001, 1.31)	*0.819 *
PRLR	**0.461 **	(0.00001, 0.96)	*0.318 *
CD97	*0.814 *	(0.70, 0.93)	**0.021 **

PRDX5	*1.143 *	(1.02, 1.27)	**0.041 **
MIF	*1.202 *	(1.02, 1.38)	**0.045 **
IFNGR2	*1.225 *	(1.10, 1.35)	**0.004 **
SDCBP	*1.244 *	(1.01, 1.48)	**0.043 **
NFX1	*1.251 *	(1.07, 1.43)	**0.016 **
CD74	*1.258 *	(1.09, 1.43)	**0.011 **
IL10RA	*1.279 *	(1.12, 1.44)	**0.007 **
BCL6	*1.315 *	(1.00, 1.63)	**0.049 **
CCL28	*1.358 *	(1.00, 1.72)	**0.049 **
NFE2L1	*1.360 *	(1.17, 1.55)	**0.003 **
EPOR	*1.362 *	(1.04, 1.69)	**0.042 **
LTB4R	*1.382 *	(1.13, 1.63)	**0.009 **
CSF3R	*1.412 *	(1.12, 1.70)	**0.013 **
TLR4	*1.441 *	(1.19, 1.70)	**0.004 **
CSF2RA	*1.740 *	(1.26, 2.22)	**0.005 **
IL18	*1.961 *	(0.95, 2.97)	**0.038 **
IL36G	**2.064 **	(0.36, 3.76)	*0.118 *
IL37	**2.084 **	(0.00001, 4.50)	*0.208 *
MEFV	**2.134 **	(0.00001, 4.68)	*0.275 *
PPBP	**2.163 **	(0.81, 3.52)	*0.087 *
CCL23	**2.249 **	(0.29, 4.21)	*0.139 *
CXCL10	**2.447 **	(0.04, 4.85)	*0.130 *
TLR9	**3.076 **	(0.06, 6.09)	*0.062 *
SIGLEC1	**3.352 **	(0.00001, 6.98)	*0.088 *

Thirty genes showed alteration at least 2-fold or statistical significance (*P* < 0.05, unpaired *t*-test).

**Table 3:** Genes differentially expressed in adipose tissue treated with nonobese preeclampsia sera (*n* = 5) versus matched control sera (*n* = 5).

Functional categories	Genes	Reference
Immune response		
Th2 predominance	BCL6	[30–32]
	CCL28	[33]
	LTB4R	[34–37]
	IL37	[38]
Th1 predominance	IL18	[7, 39–43]
	CXCL10	[44–47]
	IK	[9, 48]
Th17 predominance	IL36G	[49]
Others	MEFV	[50]
	PPBP	[51, 52]
	CCL23	[53–55]
	SIGLEC1	[56]
	CD97	[57]

Oxidative stress		
Host defense	PRDX5	[58–64]
	MIF	[65–73]
	CD74	[74–76]
	NFE2L1	[77, 78]
	CSF3R	[5, 79, 80]
Stress response	TLR4	[6, 16, 17, 28, 81–85]
	TLR9	[18, 19, 86, 87]

Insulin resistance		
Insulin sensitivity	IL10RA	[88, 89]
	TLR3	[89–94]
	FOS	[95, 96]
	PRL	[97]
Insulin resistance	IFNGR2	[98, 99]
	NFX1	[100]

Adipogenesis		
Stimulation	SDCBP	[101–104]
	OSM	[25, 26, 105–107]
Reduction	EPOR	[21–23, 108]
	CSF2RA	[24, 109]

Microarray analyses indicated that 24 upregulated genes and 6 downregulated genes in preeclampsia sera-treated adipose tissue were involved in immune response, oxidative stress, insulin resistance, and lipid metabolism. Gene expression was confirmed by RT-qPCR and independently validated.

## Abbreviations

BCL6: B-cell CLL/lymphoma 6
CCL28: Chemokine (C-C motif) ligand 28
CCL23: Chemokine (C-C motif) ligand 23
CD74: Major histocompatibility complex, class II invariant chain
CD97: CD97 molecule
CSF2RA: Colony stimulating factor 2 receptor, alpha, low-affinity (granulocyte-macrophage)
CSF3R: Colony stimulating factor 3 receptor
CXCL10: Chemokine (C-X-C motif) ligand 10
EPOR: Erythropoietin receptor
FOS: FBJ murine osteosarcoma viral oncogene homolog
IFNGR2: Interferon gamma receptor 2
IK: IK cytokine, downregulator of HLA II
IL10RA: Interleukin 10 receptor, alpha
IL18: Interleukin 18 (interferon-gamma-inducing factor)
IL36G: Interleukin 36, gamma
IL37: Interleukin 37
LTB4R: Leukotriene B4 receptor
MEFV: Mediterranean fever, also known as pyrin
MIF: Macrophage migration inhibitory factor
NFE2L1: Nuclear factor, erythroid 2-like 1
NFX1: Nuclear transcription factor, X-box binding 1
OSM: Oncostatin M
PPBP: Proplatelet basic protein (chemokine (C-X-C motif) ligand 7)
PRDX5: Peroxiredoxin 5
PRLR: Prolactin receptor
SDCBP: Syndecan binding protein (syntenin)
SIGLEC1: Sialic acid binding Ig-like lectin 1, sialoadhesin
TLR3: Toll-like receptor 3
TLR4: Toll-like receptor 4
TLR9: Toll-like receptor 9.

## References

[1] Espinoza J. (2012). Uteroplacental ischemia in early- and late-onset pre-eclampsia: a role for the fetus?. *Ultrasound in Obstetrics and Gynecology*.

[2] Kim Y. J. (2013). Pathogenesis and promising non-invasive markers for preeclampsia. *Obstetrics & Gynecology Science*.

[3] LaMarca B. (2012). Endothelial dysfunction. An important mediator in the pathophysiology of hypertension during pre-eclampsia. *Minerva Ginecologica*.

[4] Szarka A., Rigó J., Lázár L., Beko G., Molvarec A. (2010). Circulating cytokines, chemokines and adhesion molecules in normal pregnancy and preeclampsia determined by multiplex suspension array. *BMC Immunology*.

[5] Brewster J. A., Orsi N. M., Gopichandran N., Ekbote U. V., Cadogan E., Walker J. J. (2008). Host inflammatory response profiling in preeclampsia using an in vitro whole blood stimulation model. *Hypertension in Pregnancy*.

[6] Al-ofi E., Coffelt S. B., Anumba D. O. (2012). Monocyte subpopulations from pre-eclamptic patients are abnormally skewed and exhibit exaggerated responses to toll-like receptor ligands. *PLoS ONE*.

[7] El-Kabarity R. H., Naguib A. H. (2011). Serum levels of IL-18, IL-12 and TH-1/TH-2 ratio in patients with pre-eclampsia. *The Egyptian journal of immunology*.

[8] Toldi G., Rigó J., Stenczer B., Vásárhelyi B., Molvarec A. (2011). Increased prevalence of IL-17-producing peripheral blood lymphocytes in pre-eclampsia. *American Journal of Reproductive Immunology*.

[9] Nair S., Lee Y. H., Rousseau E. (2005). Increased expression of inflammation-related genes in cultured preadipocytes/stromal vascular cells from obese compared with non-obese Pima Indians. *Diabetologia*.

[10] Kopp A., Buechler C., Bala M., Neumeier M., Schölmerich J., Schäffler A. (2010). Toll-like receptor ligands cause proinflammatory and prodiabetic activation of adipocytes via phosphorylation of extracellular signal-regulated kinase and c-Jun N-terminal kinase but not interferon regulatory factor-3. *Endocrinology*.

[11] Naruse K., Suzuki Y., Nakamoto O. (2013). A Brief Review of the 2009 JSSHP Guidelines for the care and treatment of Pregnancy induced Hypertension. *Hypertension Research in Pregnancy*.

[12] Sykes L., MacIntyre D. A., Yap X. J., Teoh T. G., Bennett P. R. (2012). The Th1:Th2 dichotomy of pregnancy and preterm labour. *Mediators of Inflammation*.

[13] Ryu J.-K., Tumurbaatar M., Jin H.-R. (2012). Intracavernous delivery of freshly isolated stromal vascular fraction rescues erectile function by enhancing endothelial regeneration in the streptozotocin-induced diabetic mouse. *The Journal of Sexual Medicine*.

[14] Darmochwal-Kolarz D., Kludka-Sternik M., Tabarkiewicz J. (2012). The predominance of Th17 lymphocytes and decreased number and function of Treg cells in preeclampsia. *Journal of Reproductive Immunology*.

[15] Xie F., Hu Y., Speert D. P. (2010). Toll-like receptor gene polymorphisms and preeclampsia risk: a case-control study and data synthesis. *Hypertension in Pregnancy*.

[16] Bernardi F. C. B., Felisberto F., Vuolo F. (2012). Oxidative damage, inflammation, and toll-like receptor 4 pathway are increased in preeclamptic patients: a case-control study. *Oxidative Medicine and Cellular Longevity*.

[17] Karki R., Igwe O. J. (2013). Toll-like receptor 4-mediated nuclear factor kappa B activation is essential for sensing exogenous oxidants to propagate and maintain oxidative/nitrosative cellular stress. *PLoS ONE*.

[18] Goulopoulou S., Matsumoto T., Bomfim G. F., Webb R. C. (2012). Toll-like receptor 9 activation: a novel mechanism linking placenta-derived mitochondrial DNA and vascular dysfunction in pre-eclampsia. *Clinical Science*.

[19] Khazen W., M'Bika J. P., Collinet M. (2007). Differentiation-dependent expression of interferon gamma and toll-like receptor 9 in 3T3-F442A adipocytes. *Biochimie*.

[20] Feig D. S., Shah B. R., Lipscombe L. L. (2013). Preeclampsia as a risk factor for diabetes: a population-based cohort study. *PLoS Medicine*.

[21] Teng R., Gavrilova O., Suzuki N. (2011). Disrupted erythropoietin signalling promotes obesity and alters hypothalamus proopiomelanocortin production. *Nature Communications*.

[22] Hu M.-C., Shi M., Cho H. J. (2013). The erythropoietin receptor is a downstream effector of Klotho-induced cytoprotection. *Kidney International*.

[23] Wang L., Teng R., Di L. (2013). PPARa and sirt1 mediate erythropoietin action in increasing metabolic activity and browning of white adipocytes to protect against obesity and metabolic disorders. *Diabetes*.

[24] Kim D.-H., Sandoval D., Reed J. A. (2008). The role of GM-CSF in adipose tissue inflammation. *The American Journal of Physiology—Endocrinology and Metabolism*.

[25] White U. A., Stewart W. C., Stephens J. M. (2011). Gp130 cytokines exert differential patterns of crosstalk in adipocytes both in vitro and in vivo. *Obesity*.

[26] Miyaoka Y., Tanaka M., Naiki T., Miyajima A. (2006). Oncostatin M inhibits adipogenesis through the RAS/ERK and STAT5 signaling pathways. *The Journal of Biological Chemistry*.

[27] Sitras V., Paulssen R. H., Grønaas H. (2009). Differential placental gene expression in severe preeclampsia. *Placenta*.

[28] Fusaru A. M., Stǎnciulescu C. E., Şurlin V. (2012). Role of innate immune receptors TLR2 and TLR4 as mediators of the inflammatory reaction in human visceral adipose tissue. *Romanian Journal of Morphology and Embryology*.

[29] Brenner C., Simmonds R. E., Wood S., Rose V., Feldmann M., Turner J. (2012). TLR signalling and adapter utilization in primary human *in vitro* differentiated adipocytes. *Scandinavian Journal of Immunology*.

[30] Huang C., Hatzi K., Melnick A. (2013). Lineage-specific functions of Bcl-6 in immunity and inflammation are mediated by distinct biochemical mechanisms. *Nature Immunology*.

[31] Ohtsuka Y., Arima M., Fujimura L. (2005). Bcl6 regulates Th2 type cytokine productions by mast cells activated by Fc*ε*RI/IgE cross-linking. *Molecular Immunology*.

[32] Nishizawa H., Ota S., Suzuki M. (2011). Comparative gene expression profiling of placentas from patients with severe pre-eclampsia and unexplained fetal growth restriction. *Reproductive Biology and Endocrinology*.

[33] Ezzat M. H. M., Sallam M. A., Shaheen K. Y. A. (2009). Serum mucosa-associated epithelial chemokine (MEC/CCL28) in atopic dermatitis: a specific marker for severity. *International Journal of Dermatology*.

[34] Miyahara N., Miyahara S., Takeda K., Gelfand E. W. (2006). Role of the LTB4/BLT1 pathway in allergen-induced airway hyperresponsiveness and inflammation. *Allergology International*.

[35] Hirata K., Katayama K., Nakajima A., Takada K., Kamisaki Y., Wada K. (2012). Role of leukotriene B4 receptor signaling in human preadipocyte differentiation. *Biochemical and Biophysical Research Communications*.

[36] Jung S. H., Saxena A., Kaur K. (2013). The role of adipose tissue-associated macrophages and T lymphocytes in the pathogenesis of inflammatory bowel disease. *Cytokine*.

[37] Amaral L. M., Pinheiro L. C., Guimaraes D. A. (2013). Antihypertensive effects of inducible nitric oxide synthase inhibition in experimental pre-eclampsia. *Journal of Cellular and Molecular Medicine*.

[38] Banchereau J., Pascual V., O'Garra A. (2012). From IL-2 to IL-37: the expanding spectrum of anti-inflammatory cytokines. *Nature Immunology*.

[39] Huang X., Huang H., Dong M., Yao Q., Wang H. (2005). Serum and placental interleukin-18 are elevated in preeclampsia. *Journal of Reproductive Immunology*.

[40] Skurk T., Kolb H., Müller-Scholze S., Röhrig K., Hauner H., Herder C. (2005). The proatherogenic cytokine interleukin-18 is secreted by human adipocytes. *European Journal of Endocrinology*.

[41] Venkatachalam K., Prabhu S. D., Reddy V. S., Boylston W. H., Valente A. J., Chandrasekar B. (2009). Neutralization of interleukin-18 ameliorates ischemia/reperfusion-induced myocardial injury. *Journal of Biological Chemistry*.

[42] Cruz C. M., Rinna A., Forman H. J., Ventura A. L. M., Persechini P. M., Ojcius D. M. (2007). ATP activates a reactive oxygen species-dependent oxidative stress response and secretion of proinflammatory cytokines in macrophages. *The Journal of Biological Chemistry*.

[43] Stienstra R., Joosten L. A. B., Koenen T. (2010). The inflammasome-mediated caspase-1 activation controls adipocyte differentiation and insulin sensitivity. *Cell Metabolism*.

[44] Herder C., Hauner H., Kempf K., Kolb H., Skurk T. (2007). Constitutive and regulated expression and secretion of interferon-*γ*-inducible protein 10 (IP-10/CXCL10) in human adipocytes. *International Journal of Obesity*.

[45] Williams R., Yao H., Peng F., Yang Y., Bethel-Brown C., Buch S. (2010). Cooperative induction of CXCL10 involves NADPH oxidase: implications for HIV dementia. *Glia*.

[46] Michalec L., Choudhury B. K., Postlethwait E. (2002). CCL7 and CXCL10 orchestrate oxidative stress-induced neutrophilic lung inflammation. *Journal of Immunology*.

[47] Boij R., Svensson J., Nilsson-Ekdahl K. (2012). Biomarkers of coagulation, inflammation, and angiogenesis are independently associated with preeclampsia. *The American Journal of Reproductive Immunology*.

[48] Krief P., Augery-Bourget Y., Plaisance S. (1994). A new cytokine (IK) down-regulating HLA class II: monoclonal antibodies, cloning and chromosome localization. *Oncogene*.

[49] Carrier Y., Ma H.-L., Ramon H. E. (2011). Inter-regulation of Th17 cytokines and the IL-36 cytokines in vitro and in vivo: implications in psoriasis pathogenesis. *The Journal of Investigative Dermatology*.

[50] Standing A., Omoyinmi E., Brogan P. (2013). Gene hunting in autoinflammation. *Clinical and Translational Allergy*.

[51] Matsubara J., Honda K., Ono M. (2011). Reduced plasma level of CXC chemokine ligand 7 in patients with pancreatic cancer. *Cancer Epidemiology, Biomarkers and Prevention*.

[52] Gleissner C. A., von Hundelshausen P., Ley K. (2008). Platelet chemokines in vascular disease. *Arteriosclerosis, Thrombosis, and Vascular Biology*.

[53] Poposki J. A., Uzzaman A., Nagarkar D. R. (2011). Increased expression of the chemokine CCL23 in eosinophilic chronic rhinosinusitis with nasal polyps. *The Journal of Allergy and Clinical Immunology*.

[54] Kim C.-S., Kang J.-H., Cho H.-R. (2011). Potential involvement of CCL23 in atherosclerotic lesion formation/progression by the enhancement of chemotaxis, adhesion molecule expression, and MMP-2 release from monocytes. *Inflammation Research*.

[55] Mäkikallio K., Kaukola T., Tuimala J., Kingsmore S. F., Hallman M., Ojaniemi M. (2012). Umbilical artery chemokine CCL16 is associated with preterm preeclampsia and fetal growth restriction. *Cytokine*.

[56] O'Neill A. S. G., van Den Berg T. K., Mullen G. E. D. (2013). Sialoadhesin—a macrophage-restricted marker of immunoregulation and inflammation. *Immunology*.

[57] Safaee M., Clark A. J., Oh M. C. (2013). Overexpression of CD97 confers an invasive phenotype in glioblastoma cells and is associated with decreased survival of glioblastoma patients. *PLoS ONE*.

[58] Ishii T., Warabi E., Yanagawa T. (2012). Novel roles of peroxiredoxins in inflammation, cancer and innate immunity. *Journal of Clinical Biochemistry and Nutrition*.

[59] Huh J. Y., Kim Y., Jeong J. (2012). Peroxiredoxin 3 is a key molecule regulating adipocyte oxidative stress, mitochondrial biogenesis, and adipokine expression. *Antioxidants and Redox Signaling*.

[60] Graves J. A., Metukuri M., Scott D., Rothermund K., Prochownik E. V. (2009). Regulation of reactive oxygen species homeostasis by peroxiredoxins and c-Myc. *The Journal of Biological Chemistry*.

[61] Miyamoto N., Izumi H., Miyamoto R. (2011). Quercetin induces the expression of peroxiredoxins 3 and 5 via the Nrf2/NRF1 transcription pathway. *Investigative Ophthalmology and Visual Science*.

[62] Sawicki G., Dakour J., Morrish D. W. (2003). Functional proteomics of neurokinin B in the placenta indicates a novel role in regulating cytotrophoblast antioxidant defences. *Proteomics*.

[63] Kumar P., Luo Y., Tudela C., Alexander J. M., Mendelson C. R. (2013). The c-Myc-regulated microRNA-17~92 (miR-17~92) and miR-106a~363 clusters target hCYP19A1 and hGCM1 to inhibit human trophoblast differentiation. *Molecular and Cellular Biology*.

[64] Brownbill P., Bell N. J., Woods R. J., Lowry P. J., Page N. M., Sibley C. P. (2003). Neurokinin B is a paracrine vasodilator in the human fetal placental circulation. *The Journal of Clinical Endocrinology and Metabolism*.

[65] Bruchfeld A., Carrero J. J., Qureshi A. R. (2009). Elevated serum macrophage migration inhibitory factor (MIF) concentrations in chronic kidney disease (CKD) are associated with markers of oxidative stress and endothelial activation. *Molecular Medicine*.

[66] Rao F., Deng C. Y., Zhang Q. H. (2013). Involvement of Src tyrosine kinase and protein kinase C in the expression of macrophage migration inhibitory factor induced by H_2_O_2_ in HL-1 mouse cardiac muscle cells. *Brazilian Journal of Medical and Biological Research*.

[67] Tuyet Nguyen M., Lue H., Kleemann R. (2003). The cytokine macrophage migration inhibitory factor reduces pro-oxidative stress-induced apoptosis. *Journal of Immunology*.

[68] Cardaropoli S., Paulesu L., Romagnoli R. (2012). Macrophage migration inhibitory factor in fetoplacental tissues from preeclamptic pregnancies with or without fetal growth restriction. *Clinical and Developmental Immunology*.

[69] Todros T., Bontempo S., Piccoli E. (2005). Increased levels of macrophage migration inhibitory factor (MIF) in preeclampsia. *European Journal of Obstetrics Gynecology and Reproductive Biology*.

[70] Rolfo A., Giuffrida D., Nuzzo A. M. (2013). Pro-inflammatory profile of preeclamptic placental mesenchymal stromal cells: new insights into the etiopathogenesis of preeclampsia. *PLoS ONE*.

[71] Ikeda D., Sakaue S., Kamigaki M. (2008). Knockdown of macrophage migration inhibitory factor disrupts adipogenesis in 3T3-L1 cells. *Endocrinology*.

[72] Atsumi T., Cho Y.-R., Leng L. (2007). The proinflammatory cytokine macrophage migration inhibitory factor regulates glucose metabolism during systemic inflammation. *Journal of Immunology*.

[73] Skurk T., Herder C., Kräft I., Müller-Scholze S., Hauner H., Kolb H. (2005). Production and release of macrophage migration inhibitory factor from human adipocytes. *Endocrinology*.

[74] Beswick E. J., Reyes V. E. (2009). CD74 in antigen presentation, inflammation, and cancers of the gastrointestinal tract. *World Journal of Gastroenterology*.

[75] Starlets D., Gore Y., Binsky I. (2006). Cell-surface CD74 initiates a signaling cascade leading to cell proliferation and survival. *Blood*.

[76] Savaskan N. E., Fingerle-Rowson G., Buchfelder M., Eyüpoglu I. Y. (2012). Brain miffed by macrophage migration inhibitory factor. *International Journal of Cell Biology*.

[77] Oh D. H., Rigas D., Cho A., Chan J. Y. (2012). Deficiency in the nuclear-related factor erythroid 2 transcription factor (Nrf1) leads to genetic instability. *The FEBS Journal*.

[78] Rim J. S., Kozak L. P. (2002). Regulatory motifs for CREB-binding protein and Nfe212 transcription factors in the upstream enhancer of the mitochondrial uncoupling protein 1 gene. *The Journal of Biological Chemistry*.

[79] Kojima H., Otani A., Oishi A., Makiyama Y., Nakagawa S., Yoshimura N. (2011). Granulocyte colony-stimulating factor attenuates oxidative stress-induced apoptosis in vascular endothelial cells and exhibits functional and morphologic protective effect in oxygen-induced retinopathy. *Blood*.

[80] Tanaka M., Kikuchi H., Ishizu T. (2006). Intrathecal upregulation of granulocyte colony stimulating factor and its neuroprotective actions on motor neurons in amyotrophic lateral sclerosis. *Journal of Neuropathology and Experimental Neurology*.

[81] Mkaddem S. B., Bens M., Vandewalle A. (2010). Differential activation of Toll-like receptor-mediated apoptosis induced by hypoxia. *Oncotarget*.

[82] Suliman H. B., Welty-Wolf K. E., Carraway M. S., Schwartz D. A., Hollingsworth J. W., Piantadosi C. A. (2005). Toll-like receptor 4 mediates mitochondrial DNA damage and biogenic responses after heat-inactivated *E. coli*. *The FASEB Journal*.

[83] Nativel B., Marimoutou M., Thon-Hon V. G. (2013). Soluble HMGB1 is a novel adipokine stimulating IL-6 secretion through RAGE receptor in SW872 preadipocyte cell line: contribution to chronic inflammation in fat tissue. *PLoS ONE*.

[84] Naruse K., Sado T., Noguchi T. (2012). Peripheral RAGE (receptor for advanced glycation endproducts)-ligands in normal pregnancy and preeclampsia: novel markers of inflammatory response. *Journal of Reproductive Immunology*.

[85] Resi V., Basu S., Haghiac M. (2012). Molecular inflammation and adipose tissue matrix remodeling precede physiological adaptations to pregnancy. *The American Journal of Physiology—Endocrinology and Metabolism*.

[86] Fűri I., Sipos F., Germann T. M. (2013). Epithelial toll-like receptor 9 signaling in colorectal inflammation and cancer: clinico-pathogenic aspects. *World Journal of Gastroenterology*.

[87] Ding Z., Liu S., Wang X., Khaidakov M., Dai Y., Mehta J. L. (2013). Oxidant stress in mitochondrial DNA damage, autophagy and inflammation in atherosclerosis. *Scientific Reports*.

[88] Turner J. J. O., Foxwell K. M., Kanji R. (2010). Investigation of nuclear factor-*κ*B inhibitors and interleukin-10 as regulators of inflammatory signalling in human adipocytes. *Clinical and Experimental Immunology*.

[89] Chatterjee P., Chiasson V. L., Kopriva S. E. (2011). Interleukin 10 deficiency exacerbates toll-like receptor 3-induced preeclampsia-like symptoms in mice. *Hypertension*.

[90] Chatterjee P., Weaver L. E., Doersch K. M. (2012). Placental toll-like receptor 3 and toll-like receptor 7/8 activation contributes to preeclampsia in humans and mice. *PLoS ONE*.

[91] Yu L., Yan K., Liu P. (2014). Pattern recognition receptor-initiated innate antiviral response in mouse adipose cells. *Immunology and Cell Biology*.

[92] Franchini M., Monnais E., Seboek D. (2010). Insulin resistance and increased lipolysis in bone marrow derived adipocytes stimulated with agonists of toll-like receptors. *Hormone and Metabolic Research*.

[93] Patel A. K., Hackam A. S. (2013). Toll-like receptor 3 (TLR3) protects retinal pigmented epithelium (RPE) cells from oxidative stress through a STAT3-dependent mechanism. *Molecular Immunology*.

[94] Tinsley J. H., Chiasson V. L., Mahajan A., Young K. J., Mitchell B. M. (2009). Toll-like receptor 3 activation during pregnancy elicits preeclampsia-like symptoms in rats. *American Journal of Hypertension*.

[95] Jones M. R., Chazenbalk G., Xu N. (2012). Steroidogenic regulatory factor FOS is underexpressed in polycystic ovary syndrome (PCOS) adipose tissue and genetically associated with PCOS susceptibility. *The Journal of Clinical Endocrinology and Metabolism*.

[96] MacKenzie R. M., Sandrim V. C., Carty D. M. (2012). Endothelial FOS expression and pre-eclampsia. *BJOG: An International Journal of Obstetrics and Gynaecology*.

[97] Carré N., Binart N. (2014). Prolactin and adipose tissue. *Biochimie*.

[98] Vidal C., Bermeo S., Li W., Huang D., Kremer R., Duque G. (2012). Interferon gamma inhibits adipogenesis in vitro and prevents marrow fat infiltration in oophorectomized mice. *Stem Cells*.

[99] Dahlstrøm B., Esbensen Y., Vollan H., Øian P., Bukholm G. (2010). Genome profiles in maternal blood during early onset preeclampsia and towards term. *Journal of Perinatal Medicine*.

[100] Morris D. L., Cho K. W., Delproposto J. L. (2013). Adipose tissue macrophages function as antigen-presenting cells and regulate adipose tissue CD4+ T cells in mice. *Diabetes*.

[101] Sarkar D., Boukerche H., Su Z.-Z., Fisher P. B. (2008). mda-9/Syntenin: more than just a simple adapter protein when it comes to cancer metastasis. *Cancer Research*.

[102] Qian X.-L., Li Y.-Q., Yu B. (2013). Syndecan binding protein (SDCBP) is overexpressed in estrogen receptor negative breast cancers, and is a potential promoter for tumor proliferation. *PLoS ONE*.

[103] Stacker S. A., Runting A. S., Caesar C. (2000). The 3T3-L1 fibroblast to adipocyte conversion is accompanied by increased expression of angiopoietin-1, a ligand for tie2. *Growth Factors*.

[104] Ju R., Zhuang Z. W., Zhang J. (2014). Angiopoietin-2 secretion by endothelial cell exosomes: regulation by the phosphatidylinositol 3-kinase (PI3K)/Akt/endothelial nitric oxide synthase (eNOS) and syndecan-4/syntenin pathways. *The Journal of Biological Chemistry*.

[105] Rega G., Kaun C., Demyanets S. (2007). Vascular endothelial growth factor is induced by the inflammatory cytokines interleukin-6 and oncostatin m in human adipose tissue in vitro and in murine adipose tissue in vivo. *Arteriosclerosis, Thrombosis, and Vascular Biology*.

[106] Rega G., Kaun C., Weiss T. W. (2005). Inflammatory cytokines interleukin-6 and oncostatin M induce plasminogen activator inhibitor-1 in human adipose tissue. *Circulation*.

[107] Lee G., Kil G., Kwon J., Kim S., Yoo J., Shin J. (2009). Oncostatin M as a target biological molecule of preeclampsia. *The Journal of Obstetrics and Gynaecology Research*.

[108] Elliott S., Sinclair A. M. (2012). The effect of erythropoietin on normal and neoplastic cells. *Biologics: Targets & Therapy*.

[109] Moldenhauer L. M., Keenihan S. N., Hayball J. D., Robertson S. A. (2010). GM-CSF is an essential regulator of T cell activation competence in uterine dendritic cells during early pregnancy in mice. *The Journal of Immunology*.

