# Supplementary material for: Involvement of Visceral Adipose Tissue in Immunological Modulation of Inflammatory Cascade in Preeclampsia
Source: Mediators Inflamm. 2015 May 31;2015:325932. doi: 10.1155/2015/325932 (PMC4458290; doi:10.1155/2015/325932)
Supplement: Supplementary file 1 — Supplementary data shows the detailed explanation of genes differentially expressed in adipose tissue treated with nonobese preeclampsia sera versus matched control sera. [file 325932.f1.pdf]

Supplementary data

This data shows the detailed explanation of genes differentially expressed in adipose tissue treated with nonobese preeclampsia sera vs. matched control sera.

Up-regulated genes

PRDX5: peroxiredoxin 5

This gene encodes a member of the peroxiredoxin family of antioxidant enzymes, possessing thioredoxin or glutathione peroxidase (http://www.ncbi.nlm.nih.gov/gene/25824). Peroxiredoxins reduce hydrogen peroxide and alkyl hydroperoxides, function to scavenge reactive oxygen species (ROS), and therefore protect cells from oxidative insults. Peroxiredoxins are also associated with inflammation-related biological reactions such as tissue repair, oxidative stress, parasite infection and tumor progression [30]. Peroxiredoxins maintain normal characteristics of adipocytes, possibly through regulation of adipocyte oxidative stress, mitochondrial biogenesis, adipokine expression, impaired glucose tolerance and insulin resistance [31]. Increased oxidative stress and mitochondrial dysfunction in adipocytes likely contribute to adipokine dysregulation, inflammation, and insulin resistance [31]. PRDX5 (peroxiredoxin 5) plays an antioxidant protective role in different tissues under normal conditions and during inflammatory processes. This protein can inhibit the accumulation of fat in adipocytes through suppression of oxidative stress and maintain normal characteristics of adipocytes.

PRDX5 levels appear to be highly dependent on inflammatory cytokines tumor necrosis factor (TNF)-alpha, interleukin (IL)-1beta and interferon (IFN)-gamma, lipopolysaccharide (LPS)-Toll-like receptor (TLR)4 signaling, transcription factors v-ets avian erythroblastosis virus E26 oncogene homolog 1 (Ets1) and Ets2 [30], v-myc avian myelocytomatosis viral oncogene homolog (c-Myc) [32], nuclear respiratory factor 1 (NRF1) [33] and Neurokinin B (NKB) [34]. c-Myc stimulates trophoblast proliferation and inhibits cell differentiation through overexpression of members of the microRNA, suggesting that c-Myc is a mediator of some pathophysiological features of preeclampsia [35]. NRF1 can activate the expression of some key metabolic genes regulating cellular growth, mitochondrial target genes, the

transcription of nuclear genes encoding respiratory complex subunits, heme biosynthesis, and mitochondrial DNA transcription and replication (http://www.ncbi.nlm.nih.gov/gene/4899). NKB is a member of the tachykinin family of neurotransmitters, including substance P and neurokinins [36]. The placenta is the main site of NKB and its receptor expression. NKB is overexpressed in preeclampsia, which causes hypertension in rats, suggesting that it may be involved in the pathogenesis of preeclampsia [34]. NKB also play an essential role in antioxidant defenses. Up-regulation of the NKB-dependent PRDX5 expression may prevent preeclampsia-induced oxidative stress.

MIF: macrophage migration inhibitory factor

This gene encodes a lymphokine involved in cell-mediated immunity, immunoregulation, and inflammation (http://www.ncbi.nlm.nih.gov/gene/4282). It plays a role in the regulation of macrophage function in the host immune response or the host defense against several pathogens, and also potently inhibits cell apoptosis. This lymphokine may indicate an additional role in integrin and cell survival signaling pathways. MIF is associated with the expression level of intercellular adhesion molecule 1 (ICAM-1) molecule, which is typically expressed on endothelial cells and cells of the immune system [37].

MIF plays an important pathogenetic role in chronic kidney disease patients [37]. Oxidative stress such as hydrogen peroxide (H_2O_2) upregulates MIF expression through Src tyrosine kinase and protein kinase C (PKC)-dependent mechanisms [38]. MIF is also associated with a marker of oxidative stress, 8-hydroxy-2-deoxyguanosine (8-OHdG), suggesting that this lymphokine is a modulator of oxidative stress-induced apoptosis [37,39]. One hand, increased MIF levels are correlated with oxidative stress. On the other hand, the cytokine MIF exhibits an anti-inflammatory activity and regulates cell survival [39]. MIF actually controls survival through the Akt pathway encompassing signaling through the MIF receptor CD74 and the upstream kinases Src and phosphatidyl inositol 3 kinase (PI3K).

MIF acts in reproductive functions and is specifically involved in physiological and pathological processes in pregnancy [40]. Aberrant expression of many pro-inflammatory cytokines was observed in preeclamptic placenta. Significant increase of MIF maternal serum levels

was observed in preeclampsia patients when compared with controls, supporting the role of inflammation in the pathogenesis of this disease [40,41]. Involvement of MIF in the pathogenesis of preeclampsia was also confirmed by experiments with physiological chorionic villous explants [40,42].

Furthermore, MIF stimulates differentiation of preadipocytes through promotion of mitotic clonal expansion, demonstrating that MIF is associated with adipogenesis [43]. A response of the host against the systemic inflammation is MIF-dependent hyperglycemia and resistance to the action of insulin [44]. Adipocytes of women affected by preeclampsia produce substantial amounts of MIF, which in turn stimulates macrophage infiltration of adipose tissue [45].

IFNGR2: interferon gamma receptor 2

Human interferon-gamma receptor is a heterodimer of IFNGR1 and IFNGR2 that utilizes the Janus kinase (JAK) - signal transducer and activator of transcription (STAT) signaling pathway (http://www.ncbi.nlm.nih.gov/gene/3460). The IFNGR2 gene encodes the non-ligand-binding beta chain of the IFN-gamma receptor. IFN-gamma is a key molecule of T helper 1 (Th1)-immune response. IFN-gamma regulates signaling by Toll-like receptors, inflammation, inflammatory cytokines, or anti-inflammatory cytokines.

Clinicians know that elevation of the blood glucose level is a causal adverse effect of treatment with IFN. IFN-gamma induces insulin resistance in adipocytes. IFN-gamma also inhibits adipocyte differentiation and adipogenesis [46]. Dahlstrøm et al. reported that placental IFNGR2 expression was down-regulated in early onset preeclampsia [47], but up-regulates in adipocytes of women affected by preeclampsia.

SDCBP: syndecan binding protein (syntenin)

The protein encoded by this gene was initially identified as a molecule linking syndecan-mediated signaling to the cytoskeleton (http://www.ncbi.nlm.nih.gov/gene/6386). Syntenin is involved in several actin-polarized processes, such as cell migration, cell-surface targeting, organization of protein complexes in the plasma membranes, intracellular trafficking, immune synapse formation, synaptic transmission, axonal

outgrowth, regulation of B-cell development, and cancer metastasis [48]. SDCBP contributes to cell growth through regulating the G1/S checkpoint machinery during the cell cycle [49]. Its binding protein, syndecan-1, is a cell surface heparan sulphate proteoglycan, which binds to the extracellular matrix and many mediators such as growth factors and antithrombin III. Syndecan-1 can modulate leukocyte recruitment, microbial attachment and entry, growth factor binding, host defense mechanisms, anticoagulation, angiogenesis, matrix remodeling, cytoskeletal-membrane organization, cell adhesion, tumor cell proliferation and invasion. Syndecan-1 in chorionic villi also plays a potential role in feto-maternal inter-communication between the embryo and the extracellular matrix (ECM) of decidua.

The mode of expression of syntenin has never been established in adipocytes. Sera from preeclamptic patients can induce the expression of syntenin in adipocytes. Syntenin is involved in secretion of exosomal angiopoietin, which is related to the regulation of inflammation, vascular integrity and cellular differentiation in adipose development [50,51]. Syntenin in adipocytes may play important roles in vascular growth, stabilization, local inflammation and adipogenesis in patients with preeclampsia.

NFX1: nuclear transcription factor, X-box binding 1

The protein encoded by this gene is a transcriptional repressor capable of binding to the highly conserved X box motif element of HLA-DRA and other major histocompatibility complex (MHC) class II genes (http://www.ncbi.nlm.nih.gov/gene/4799). The NFX1 protein regulates the duration of an inflammatory response by modulating IFN-gamma-induced MHC class II molecules. NFX1 is implicated in a feedback loop to limit the immune response following infection and inflammation.

The activation of leukocytes due to a low-grade systemic or adipose tissue inflammation contributes to metabolic disease [52]. Macrophages in adipose tissue express functional MHC class II-restricted antigens, which promote the proliferation of IFN-gamma-producing $CD4^+$ T cells in adipose tissue [52]. Functional MHC class II antigen might develop adipose inflammation and insulin resistance.

CD74: major histocompatibility complex, class II invariant chain

The protein encoded by this gene associates with MHC class II and is an important chaperone that regulates antigen presentation for immune response (http://www.ncbi.nlm.nih.gov/gene/972) [53]. In addition, CD74 serves as cell surface receptor for the cytokine MIF [54]. MIF regulates essential cellular systems such as redox balance, oxidative stress, DNA synthesis, cell division, p53-mediated senescence and apoptosis, NF-kappaB activation and up-regulation of BCL-XL expression and hypoxia inducible factor 1 (HIF-1) via CD74-dependent chemokine (C-X-C motif) receptor-mediated pathways [54,55]. The D-dopachrome tautomerase (DDT), a novel adipokine, induces IL-6 expression in preadipocytes through the CD74 pathway.

IL10RA: interleukin 10 receptor, alpha

The protein encoded by this gene is a receptor for interleukin 10 (http://www.ncbi.nlm.nih.gov/gene/3587). This protein is structurally related to interferon receptors. IL10 is an immunosuppressive cytokine that inhibits the synthesis of proinflammatory cytokines TNF-alpha and IFN-alpha made by macrophages and Th1 cells. IL-2 selectively enhances production of IL10 through activation of STAT5.

IL10 can suppress hyperphagia-related obesity associated with insulin and leptin resistance, demonstrating that it functions as a regulator of inflammatory signalling in adipocytes [56]. Chatterjee et al. reported that IL10 deficiency exacerbates TLR3-induced preeclampsia-like symptoms in mice [57].

BCL6: B-cell CLL/lymphoma 6

The protein encoded by this gene is a zinc finger transcription repressor (http://www.ncbi.nlm.nih.gov/gene/604). BCL6 is a key player in cell survival, facilitating rapid cell proliferation and tolerance of genomic damage through repressing DNA damage sensing and checkpoint genes such as ataxia telangiectasia and Rad3 related (ATR), checkpoint kinase 1 (CHEK1), TP53 and cyclin-dependent kinase inhibitor 1A (CDKN1A, also known as p21, Cip1) [58]. The forkhead box O1 (FOXO1) / B-cell CLL/lymphoma 6 (BCL6) / cyclin D2 pathway also plays a role in myogenic growth and differentiation. BCL6 negatively regulates nuclear factor (NF)-kappaB expression and modulates balanced Th1/Th2 differentiation through stimulating Th2 type

cytokine productions [59]. BCL6 can reduce pro-inflammatory signaling in macrophages. BCL6 is up-regulated in preeclampsia [60].

CCL28: chemokine (C-C motif) ligand 28

The cytokine encoded by this gene is the T cell chemoattractant (http://www.ncbi.nlm.nih.gov/gene/56477). CCL28 specifically displays chemotactic activity for resting CD4 or CD8 T cells and eosinophils. This cytokine is highly upregulated in inflammatory skin diseases, such as atopic dermatitis, probably through the selective migration and infiltration of effector/memory Th2 cells in the skin [61]. Pro-inflammatory cytokines that signal through NF-kappaB induce CCL28 expression. Up-regulation of CCL28 expression represents a shift towards Th2-type immunity.

NFE2L1: nuclear factor, erythroid 2-like 1

This gene encodes a protein that is involved in globin gene expression in erythrocytes (http://www.ncbi.nlm.nih.gov/gene/4779). NFE2L1 stimulates antioxidant response element-driven transcriptional activity, and preferentially activates a subset of oxidative stress response genes and maintains genomic integrity [62,63].

EPOR: erythropoietin receptor

The protein encoded by this gene activates JAK2 tyrosine kinase which activates RAS / mitogen-activated protein kinase 1 (MAPK), PI3K and STAT transcription factors (http://www.ncbi.nlm.nih.gov/gene/2057). EPOR has a role in erythroid cell survival, thereby promoting erythropoiesis, and tissue protection [64]. EPOR regulates energy homeostasis and mitigates oxidative damage, insulin resistance and adipogenesis via the metabolism coregulators peroxisome proliferator-activated receptor alpha (PPARalpha) and sirtuin 1 (Sirt1) [21,22,23]. PPARalpha affects the expression of target genes involved in cell proliferation, cell differentiation and in immune and inflammation responses (http://www.ncbi.nlm.nih.gov/gene/5465). Sirt1 functions as intracellular regulatory proteins with mono-ADP-ribosyltransferase activity (http://www.ncbi.nlm.nih.gov/gene/23411). EPO has shown beneficial effects in the regulation of obesity and metabolic syndrome

LTB4R: leukotriene B4 receptor

Leukotriene B4 (LTB4) is a proinflammatory lipid mediator generated from arachidonic acid through the action of 5-lipoxygenase and implicated in a wide variety of inflammatory disorders such as asthma [65]. LTB4R is a G-protein-coupled receptor for LTB4. The LTB4-LTB4R signaling pathway not only stimulates Th2 cytokine IL13 production from T cells [65], but also inhibits preadipocyte differentiation via induction of TGF-beta expression [66]. Adipocytes are a source of Th2 cytokines, including IL-13. Treatments of cytokine IL-13 to adipose tissue macrophages reduced inducible nitric oxide synthase (iNOS) expression [67]. iNOS-induced up-regulation of NO expression may have detrimental consequences to the cardiovascular system and contribute to hypertension in pregnant women. Amaral et al. reported that a selective iNOS inhibitor could exert antihypertensive effects in preeclampsia [68]. Taken together, LTB4R in adipocytes may stimulate IL13 expression and reduce iNOS expression.

CSF3R: colony stimulating factor 3 receptor

Colony stimulating factor 3 (CSF3, also known as granulocyte colony-stimulating factor (GCSF)) is a cytokine that controls the production, differentiation, and function of granulocytes (http://www.ncbi.nlm.nih.gov/gene/1441). Although CSF3 is a mediator of the preeclamptic response [5], this cytokine has a protective effect on endothelial cells against oxidative stress [69] and potentially neuroprotective effects on motor neurons [70].

TLR4: toll-like receptor 4

Toll-like receptors (TLRs) play an essential role in pathogen recognition and activation of innate immunity (http://www.ncbi.nlm.nih.gov/gene/7099). They recognize versatile pathogen-associated molecular patterns (PAMPs) and endogenous constituents called danger-associated molecular patterns (DAMPs) that mediate the production of cytokines necessary for the development of strong and effective immunity [71]. Preeclampsia monocytes are hyper-responsive to TLR ligands with respect to profound secretion of various cytokines [6]. Hyper-responsiveness is related to exacerbated progression of preeclampsia [6]. The TLR4-dependent NF-kappaB pathway upregulated in preeclampsia might generate local and systemic

inflammatory and oxidative stress responses [16]. Oxidative stress can in turn induce and maintain associated innate immune and inflammatory responses by acting mainly through a TLR4-dependent pathway [17]. The TLR4-mediated proinflammatory mediator enhances oxidative mitochondrial DNA damage, which is the initial event leading to tissue degeneration [72].

Low-grade inflammation characterized by elevated proinflammatory gene expression is a central phenomenon in the genesis of metabolic syndrome such as obesity and insulin-resistance [73]. IL6 and high mobility group box 1 (HMGB1) are major cytokines contributing to low-grade inflammation implementation and maintenance [73]. These cytokines are involved in the proinflammatory process in patients with preeclampsia [74]. HMGB1 acts as a cytokine to drive the production of inflammatory molecules through receptor for advanced glycation end-products (RAGE) and TLR2/4 [73]. Since adipocytes and infiltrating immune cells secrete pro-inflammatory adipokines and cytokines, the adipose tissue is emerged to have an essential role in the innate immunity [28]. Adipocytes are considered effector cells due to the presence of the TLRs [28]. The TLR4 signaling pathway may be enhanced in relation to inflammatory adipokines and chemokines genes in adipose tissue in women affected by obesity or preeclampsia [75].

CSF2RA: colony stimulating factor 2 receptor, alpha, low-affinity (granulocyte-macrophage)

CSF2RA controls the production, differentiation, and function of granulocytes and macrophages (http://www.ncbi.nlm.nih.gov/gene/1438). The encoded protein is a member of the cytokine family of receptors. CSF2 (also known as granulocyte-macrophage colony-stimulating factor (GM-CSF)) is synthesized by uterine epithelial cells during induction of tolerance in early pregnancy [76]. GM-CSF stimulates trophoblast cell migration. GM-CSF also influences dendritic cells and macrophage differentiation and recruitment into inflammatory sites. Dendritic cells are important for the establishment of immunological tolerance of the semiallogeneic fetus [76]. An excessive number of dendritic cells has been implicated in the impairment of trophoblast invasion in preeclampsia, suggesting that GM-CSF plays a crucial role in the pathogenesis of preeclampsia.

Furthermore, GM-CSF is related to a central action to reduce food intake and body weight, since knockout mice are more obese and hyperphagic than wild-type mice [24].

IL18: interleukin 18 (interferon-gamma-inducing factor)
The protein encoded by this gene is a member of the IL-1 family of cytokines. IL-18 is a proinflammatory cytokine with pleiotropic qualities that stimulates IFN-gamma production in Th1 cells (http://www.ncbi.nlm.nih.gov/gene/3606). IL-18 plays a role in pregnancy, labor onset and pregnant complications [77]. IL-18 acts in synergy with IL-12 or IL-15 to promote development of Th1 responses and to produce IFN-gamma from T-cells and natural killer cells [7]. Both serum and placental levels of IL-18 were significantly increased in preeclampsia as compared with control [77]. Th2 dominance in normal pregnancy shifts to Th1 dominance in preeclampsia, indicating that the Th1: Th2 ratio was higher in women with preeclampsia when compared to normal pregnant women [7]. These data suggest that preeclampsia is the Th1-type immunity disorder [7].

IL-18 is an important mediator of innate immunity, obesity, insulin resistance and strong risk factor for the development of cardiovascular disease [78]. Activation of tissue macrophages result in oxidative stress and inflammation, leading to secretion of proinflammatory cytokines such as IL18 and ischemia/reperfusion-induced injury of cardiac myocytes [79,80]. IL-18 plays critical roles in these processes. IL-18 secreted by human adipocytes also acts as a pro-atherosclerotic cytokine with insulin resistance [78].

Caspase-1 activation is associated with adipocyte differentiation and insulin resistance [81]. Caspase-1 is a cysteine protease regulated by a protein complex called the inflammasome and functions as a regulator of IL-18 activation. Elevated circulating concentrations of IL-18 are associated with obesity-related diseases.

IL36G: interleukin 36, gamma
The protein encoded by this gene is a member of the IL-1 cytokine family (http://www.ncbi.nlm.nih.gov/gene/56300). IFN-gamma, TNF-alpha, IL-1beta and Th17 cytokines stimulate the expression of IL36-gamma [82].

Th17 cells play a key role in the pathogenesis of autoimmune inflammation, including autoimmune diseases, host defense, inflammatory disease, tumorigenesis, transplant rejection and preeclampsia. Similar to pro-inflammatory cytokines, IL36 activates MAPK and NF-kappaB pathways. The IL36-gamma expression in keratinocytes can be induced by a contact hypersensitivity reaction, psoriasis, or herpes simplex virus infection.

IL37: interleukin 37

The protein encoded by this gene is a member of the IL-1 cytokine family (http://www.ncbi.nlm.nih.gov/gene/27178). IL-37 binds to IL-18 binding protein (IL18BP), an inhibitory binding protein of IL18, and inhibits the activity of IL-18. Thus, IL37 downregulates IL-18-induced inflammation [83]. IL-1 family pro-inflammatory effects are markedly suppressed by IL-37.

MEFV: Mediterranean fever, also known as pyrin

This gene encodes a protein, also known as pyrin or marenostrin, that is an important modulator of innate immunity (http://www.ncbi.nlm.nih.gov/gene/4210). MEFV interacts with the apoptotic protein ASC (apoptosis-associated speck-like protein containing a CARD, also known as PYCARD, PYD and CARD domain containing), the cytoskeletal adaptor protein PSTPIP1 (proline-serine-threonine phosphatase interacting protein 1), the inflammatory Caspase-1, certain forms of the cytosolic anchoring protein 14-3-3 and a pro-apoptotic protein SIVA1 (SIVA apoptosis-inducing factor). This gene is associated with suppression of anti-apoptotic activity and assembly of inflammasomes. MEFV is responsible for autoinflammatory diseases such as Behçet's disease, amyloidosis, ankylosing spondylitis and psoriatic juvenile idiopathic arthritis. [84].

PPBP: pro-platelet basic protein (chemokine (C-X-C motif) ligand 7)

The protein encoded by this gene is a platelet-derived growth factor that belongs to the CXC chemokine family (http://www.ncbi.nlm.nih.gov/gene/5473). CXCL7 can stimulate angiogenesis, DNA synthesis, cell mitosis, cell invasion, glycolysis, intracellular cAMP accumulation, prostaglandin E_2 secretion, and synthesis of heparanase, hyaluronic acid and sulfated glycosaminoglycan. CXCL7 binds to CXC

chemokine receptor 2 (CXCR2) on endothelium and mediates angiogenesis through activation of the Ras/Raf/MAPK- and PI3K/AKT/mTOR-induced vascular endothelial growth factor (VEGF) and heparanase expression [85]. The megakaryocyte CXCL7 also acts as a neutrophil chemoattractant [86].

CCL23: chemokine (C-C motif) ligand 23

CCL23, a member of the CC subfamily, displays chemotactic activity on resting T lymphocytes, monocytes, dendritic cells and endothelial cells via the chemokine receptor CCR1 [87] (http://www.ncbi.nlm.nih.gov/gene/6368). CCL23 stimulates the expression of adhesion molecule CD11c and matrix metalloproteinase 2 (MMP2), which results in monocyte chemotaxis and endothelial cell migration, tube formation and angiogenesis [87,88]. Since expression of CCL23 has been regulated by the Th2 cytokines IL-4 and IL-13, this cytokine is a biomarker for some inflammatory diseases including atopic dermatitis, rheumatoid arthritis and systemic sclerosis [87]. Oxidative stress stimulates the CCL23 expression from macrophages [88]. The CCL23-CCR1 immune response was up-regulated in preeclampsia [89].

CXCL10: chemokine (C-X-C motif) ligand 10

This gene encodes a chemokine of the CXC subfamily and ligand for the receptor CXCR3, which results in stimulation of monocytes, natural killer and T-cell migration (http://www.ncbi.nlm.nih.gov/gene/3627). T cell recruitment to sites of inflammation leads to an expression of IFN-gamma, which, in turn, stimulates CXCL10 secretion. This chemokine may play a role in the pro-inflammatory and anti-angiogenic properties and modulation of adhesion molecule expression through IFN-gamma-induced signaling in adipocytes and innate immune cells [90]. Oxidative stress-induced expression of CXCL10 is associated with astrocyte activation and neutrophil inflammation [91,92]. Mature adipocytes secrete several chemokines including CXCL10 [90]. The Th1-associated chemokines such as CXCL10 were significantly higher in the preeclampsia group than in controls [93].

TLR9: toll-like receptor 9

The protein encoded by this gene is a member of the TLR family which plays a fundamental role in pathogen recognition and activation of innate immunity (http://www.ncbi.nlm.nih.gov/gene/54106). TLRs recognize specific

motifs which are present in bacteria, fungi, prokaryotes and viruses. Amongst TLRs, TLR9 mounts an innate immune response through activation by bacterial or viral DNA fragments, but not vertebrate DNA, which contain unmethylated cytosine-guanine nucleotide sequences (CpGs) [94]. Mitochondrial DNA is not present in the extracellular space during health, but after cell death or organ injury, extracellular double-stranded DNA of mitochondrial DNA triggers inflammation through TLR9, because there are evolutionarily conserved similarities between bacterial DNA and mitochondrial DNA [18]. A local inflammation in atherosclerosis appears to be induced by oxidative stress-mediated damaged mitochondrial DNA [95]. TLR9 and IFN-gamma were located in differentiated and mature adipocytes [19]. It appears that preeclampsia is characterized by a combination of oxidative stress and chronic inflammation. Exaggerated placental necrosis and damaged trophoblast cells result in the release of mitochondrial DNA, which stimulates TLR9 to produce systemic maternal inflammation including adipocytes, and subsequent vascular dysfunction that may, in turn, lead to preeclampsia [18].

SIGLEC1: sialic acid binding Ig-like lectin 1, sialoadhesin
This gene encodes a member of the immunoglobulin superfamily and is a macrophage-restricted lectin-like adhesion molecule that binds glycoconjugate ligands on cell surfaces in a sialic acid-dependent manner (http://www.ncbi.nlm.nih.gov/gene/6614). High expression found on inflammatory macrophages is involved in mediating cell-cell interactions and in a variety of pathological conditions, including autoimmune inflammatory infiltrates and tumors [96].

Down-regulated genes

TLR3: toll-like receptor 3
This gene encodes TLR3 that recognizes pathogens and mediates signaling pathways important for host defense against viruses. TLR3 is abundantly expressed in placenta, and restricted to the dendritic cells (http://www.ncbi.nlm.nih.gov/gene/7098). The TLR3 ligand is a double-stranded RNA that is associated with viral infection. TLR3

stimulates the expression of multiple antiviral proteins, but often induces cell necrosis and apoptosis. This study showed that TLR3 is expressed on adipocytes. TLR3 induces the activation of pro-inflammatory immune response in adipocytes via NF-kappaB-induced expression of type I interferons (IFN-alpha/beta) [97,98]. Insulin-induced glucose uptake was decreased after activation of TLR3, suggesting that TLR3 activation is associated with insulin resistance [99]. Adipocytes exhibit innate antiviral system [98] and TLR3 protects cells from oxidative stress [100].

The expression of TLR3 mRNA and protein in placenta was increased in women with preeclampsia compared to normotensive women [97]. Maternal immune system activation via TLR3 would cause preeclampsia-like symptoms in animals [57,101].

OSM: oncostatin M

Oncostatin M (OSM) is a member of an inflammatory cytokine family that includes leukemia-inhibitory factor (LIF), granulocyte colony-stimulating factor (G-CSF), and IL-6 (http://www.ncbi.nlm.nih.gov/gene/5008). OSM-induced angiogenesis in adipose tissue is mediated through activation of VEGF signaling pathway [102]. Adipose tissue produces inflammatory mediators and plasminogen activator inhibitor type-1 (PAI-1) via OSM [103]. OSM contributes to the increased cardiovascular risk of obese patients [103]. OSM inhibits the terminal differentiation of adipocytes through the Ras/extracellular signal-regulated kinase (ERK) and STAT5 signaling pathways [25,26].

Preeclamptic placenta showed significantly increased OSM expression relative to those of the normal group [104]. OSM may cause endothelial dysfunction, leading to hypertension and proteinuria.

IK: IK cytokine, down-regulator of HLA II

IK is the IFN-gamma inhibiting cytokine and inhibits HLA Class II and HLA-DR antigens induction by IFN-gamma [105]. The role of HLA is to present peptides derived from pathogens to T cells of the host. HLA governs immune protection against pathogens. HLA-DRA is upregulated in preadipocytes of obese subjects [9]. Th1-type cytokines, like TNF-alpha, IL-2, IL-12, IFN-gamma, are overproduced in preeclampsia. Down-regulation of IK cytokine in adipose tissue may result in the overexpression of

IFN-gamma in preeclampsia.

FOS: FBJ murine osteosarcoma viral oncogene homolog

FOS dimerizes with proteins of the jun proto-oncogene (JUN) family, thereby forming the transcription factor complex AP-1 (http://www.ncbi.nlm.nih.gov/gene/2353). Expression of the FBJ murine osteosarcoma viral oncogene homolog (FOS) gene has been associated with not only regulation of cell proliferation, differentiation, and transformation, but also apoptotic cell death. Under-expression of FOS promotes insulin resistance in adipose tissue [106], and leads to impaired angiogenesis, which thereby contributes to the development of preeclampsia [107].

PRLR: prolactin receptor

This gene encodes a receptor for the anterior pituitary lactogenic hormone, prolactin, and belongs to the type I cytokine receptor family (http://www.ncbi.nlm.nih.gov/gene/5618). PRL has a role on not only lactation and reproduction but also the adipose tissue-derived energy balance [108]. PRLR is involved in the regulation of adipogenesis [108]. The full-length PRL stimulates angiogenesis. In contrast, PRL fragments that result from proteolytic cleavage of PRL have anti-angiogenic effects. Urinary PRL levels and its anti-angiogenic PRL fragments have been associated with preeclampsia disease severity.

CD97: CD97 molecule

This gene encodes a member of the epidermal growth factor seven-span transmembrane family of adhesion G-protein coupled receptors, which mediate cell-cell interactions (http://www.ncbi.nlm.nih.gov/gene/976). The encoded protein has three ligands: CD55, a negative regulator of the complement cascade, chondroitin sulfate, a component of the ECM, and the integrin alpha5beta1 [109]. CD97 plays a role in cell adhesion and migration via interactions with these ligands [109]. CD97 facilitates mobility of leukocytes into tissue and mediate tumor invasion, migration, and angiogenesis [109].
